# Clinical insights and management perspectives in sacrococcygeal teratoma: Beyond the scalpel

**DOI:** 10.1002/ccr3.9465

**Published:** 2024-10-07

**Authors:** Anish Luitel, Rasmita Poudel, Sajjad Ahmed Khan, Hiramani Pathak, Soorya Bhattarai, Nitesh Yadav, Surya Bahadur Parajuli

**Affiliations:** ^1^ Department of pediatric Surgery Birat Medical College Teaching Hospital Morang Nepal; ^2^ Department of Pathology Birat Medical College Teaching Hospital Morang Nepal; ^3^ Department of community medicine Birat Medical College Teaching Hospital Morang Nepal

**Keywords:** case report, germ cell tumor, Hansen nodule, newborn, Teratoma

## Abstract

Sacrococcygeal teratoma (SCT), a rare germ cell malignancy in newborns, necessitates prompt surgical intervention for complete resection. Long‐term follow‐up is crucial for monitoring recurrence and managing potential complications, regardless of histopathological findings, ensuring optimal outcomes and early intervention if needed.

## 1 INTRODUCTION

Sacrococcygeal teratoma (SCT) is a rare tumor yet the most common form of teratoma in newborn children. The incidence varies from 1:30000 to 1:40000. There is striking female predominance with male to female ratio 1:3–4.^1^ They are thought to arise from pluripotent cells in Hansen's nodule, located in the anterior aspect of the coccyx.^2^ It consists of tissues from all three germ layers. SCT can be diagnosed in utero or immediately during neonatal period. Prenatal diagnosis and evaluation of tumor size and growth relies on ultrasonography while doppler scan is used to assess for heart failure.^3^ In this case we present to you a female newborn diagnosed prenatally with SCT and managed successfully with complete surgical resection and regular follow up.

## 2 CASE HISTORY/EXAMINATION

A 29‐year‐old Gravida 2 Parity 1 was referred to our hospital due to the discovery of a fetal mass in the sacrococcygeal region at 31 weeks of gestation. Throughout her pregnancy, she had been taking folic acid, and her antenatal course was uncomplicated. There were no known genetic disorders or significant medical conditions in her family history. However, the case was complicated by a delay in the antenatal consultation. Although routine screening and follow‐ups were typically performed in the second trimester, this patient did not have a detailed evaluation during that period. As a result, the SCT, which is often detectable in the second trimester through routine ultrasonography, was not identified until later in the pregnancy.

During her time at our hospital, she underwent regular weekly follow‐ups, including ultrasonography (USG) to monitor the growth of the teratoma until term. At 37 weeks of gestation, she underwent a lower segment cesarean section. The procedure proceeded uneventfully, with normal intraoperative vital signs.

**FIGURE 1:**
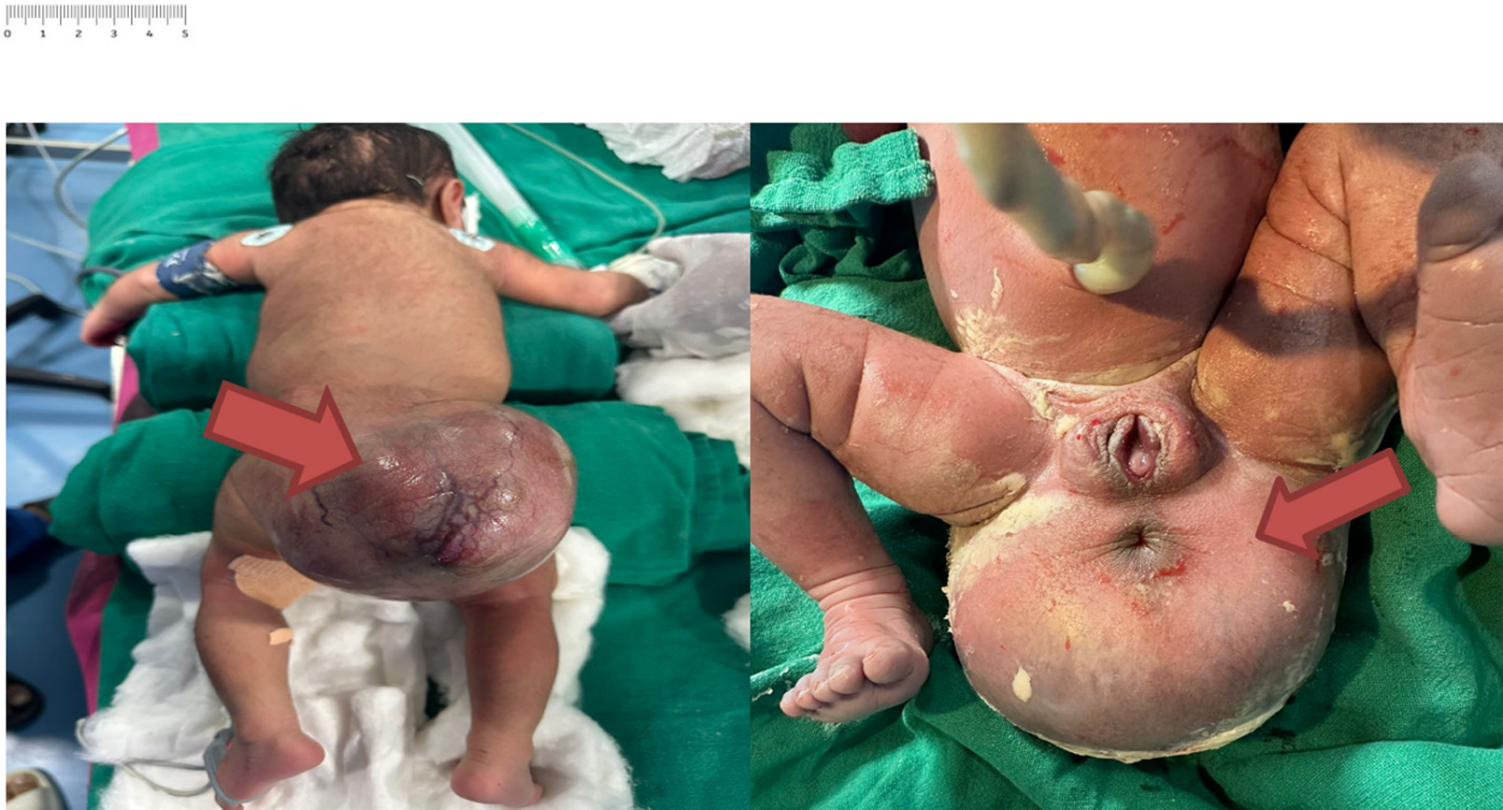
Gross appearance of newborn diagnosed with sacrococcygeal teratoma in Prone position and supine position.

The newborn, weighing 3.7 kg, displayed a heart rate of 142 beats per minute and a respiratory rate of 54 breaths per minute immediately after birth. The neonate was vigorous at birth with APGAR scores of 7/10 immediately and 8/10 at 5 min post‐birth. On thorough examination, a sizable mass classified as Altman Type IV SCT was noted, characterized by a predominantly external tumor with a significant external component but also extending internally, measuring 12.5 × 17.6 × 13.0 cm (Figure 1). The rest of the physical and systemic assessment was unremarkable.

Following initial resuscitation, the baby was promptly transferred to the Neonatal Intensive Care Unit for further evaluation and management of the SCT.

## 3 INVESTIGATIONS AND TREATMENT

The clinical diagnosis of SCT was confirmed, and a Computed Tomography (CT) scan was performed to subclassify the tumor type, assess size, extent, and vascularity. The midsagittal section of the CT scan revealed an encapsulated solid cystic mass with internal vascularity situated over the coccyx, without intrapelvic extension. Additionally, abdominal and pelvic ultrasonography (USG) was conducted to rule out associated gastrointestinal and genitourinary pathology, yielding normal findings.

**FIGURE 2:**
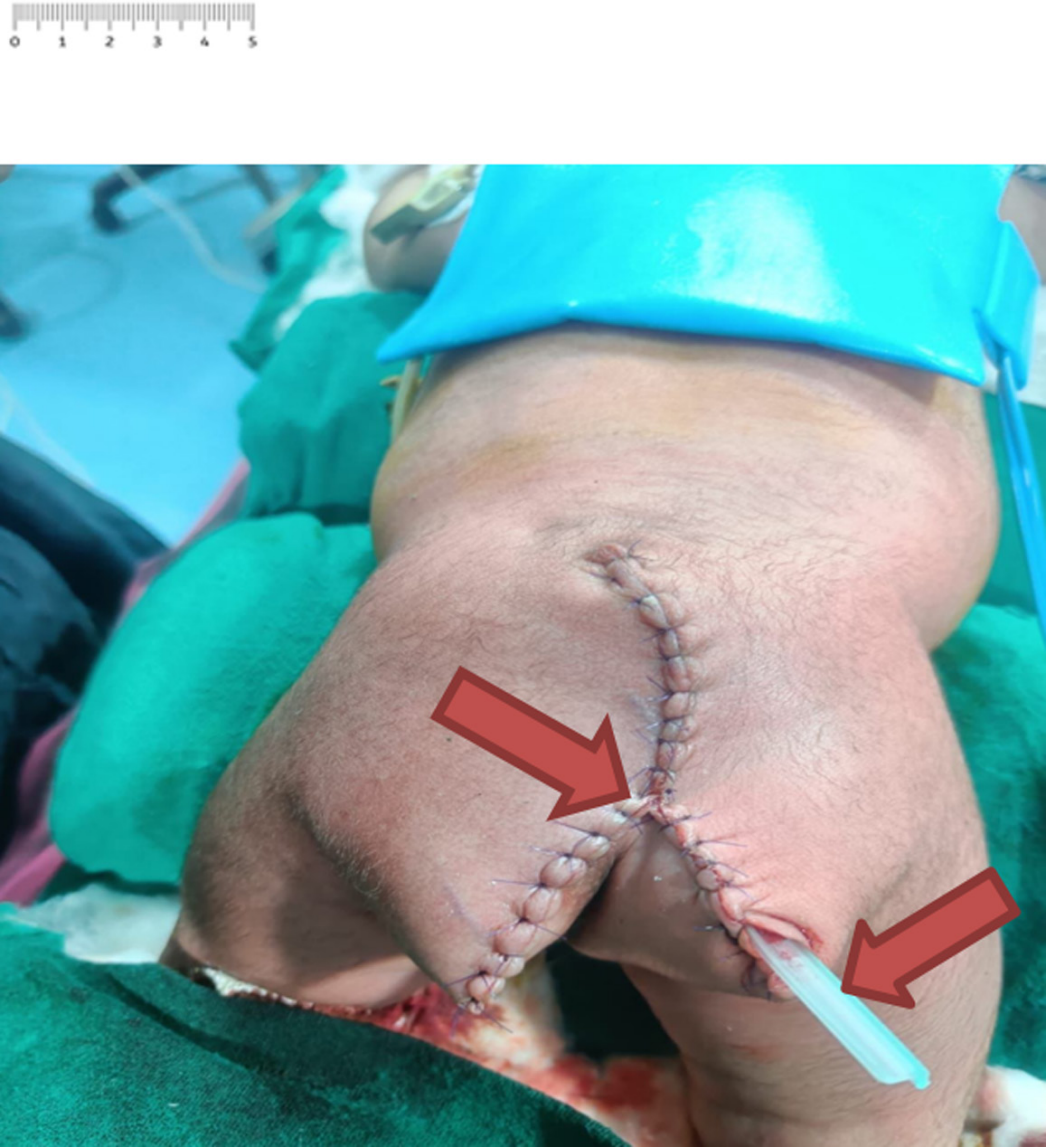
Immediate postoperative photograph with drain placed in‐situ.

On the second day of life, the infant underwent planned complete surgical resection of the SCT (Figure 2). Preoperative evaluations, including echocardiography, abdominal and pelvic USG, as well as brain and spine imaging, were normal, with no associated abnormalities detected. The alpha‐fetoprotein level was measured at 1000 ng/mL, which is considered normal for this age group.

Under general anesthesia and in the prone jackknife position, an inverted V‐shaped incision was made over the swelling. Meticulous dissection was performed, and the tumor was completely resected while preserving adjacent structures. Complete coccygectomy was performed, and it was noted that the median sacral artery was the primary blood supply to the tumor. Intraoperative blood loss was approximately 50 mL. Gross examination of the resected tumor mass revealed a globular appearance partially covered by skin, with a variegated appearance showing solid yellow and focal black areas on the cut surface.

## 4 OUTCOME AND FOLLOW‐UP

**FIGURE 3:**
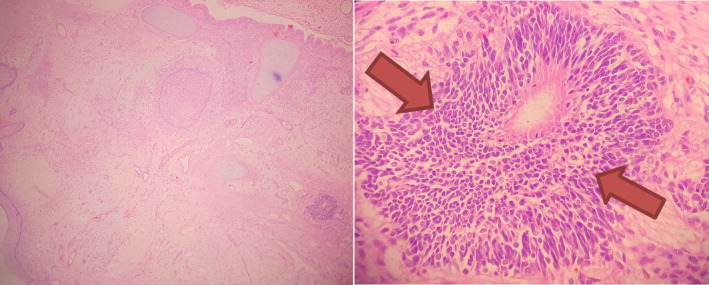
Histopathological features suggestive of immature sacrococcygeal teratoma in low magnification (40×) and high magnification under oil immersion (100×).

The tumor mass was sent for histopathological examination to establish a definitive diagnosis. Histopathology slides were stained with Hematoxylin and Eosin (H&E) and examined at low‐power magnification (40×) and high power magnification under oil immersion (100×). Histopathology slides revealed tissue lined by keratinized stratified squamous epithelium with subepithelium composed of well‐circumscribed lesions containing immature neuroepithelial elements such as rosettes, pseudorosettes, and tubules, spanning more than four low‐power fields. Additionally, glial cells, choroid plexus, and skin appendages were identified. Tumor cells exhibited round to oval nuclei with hyperchromatic nuclei and scant cytoplasm. Mucinous glands, pseudostratified ciliated columnar epithelium, cartilage, nerve bundles, and bones were also observed. Based on the gross and microscopic findings a histopatholgical diagnosis of Immature Teratoma was made (Figure 3).

The postoperative period was uneventful, and the baby was discharged home on the 4th postoperative day. The parents were counseled regarding the nature of the tumor and the necessity for regular follow‐up. Subsequently, the baby underwent examinations every 3 months until 1 year of age, including per rectal examination to assess normal anal tone. Additionally, alpha‐fetoprotein levels were monitored, showing a gradual decrease to 60 ng/dL, which is normal for the baby's age.

Currently, the plan is to continue follow‐up visits every 6 months until the child reaches 3 years of age, given the good anal tone observed and the normal alpha‐fetoprotein levels.

## 5 DISCUSSION

SCT consists of tissue from all three germ cell layers namely ectoderm, mesoderm and endoderm. It consists of hair, nail, tooth, skin, bone and cartilage, neural elements etc. Majority of SCT are benign in newborn. Contrary to what is common, histopathology report showed mature teratoma in our case. The incidence of malignant change increases with advancing age. Malignant tumors are characterized by high content of neural components. Diagnosis of tumor can be made prenatally with ultrasonography, fetal MRI scan or immediately in the postnatal period based on the classical appearance of the tumor. Majority of SCT are diagnosed in the second trimester. It is difficult to diagnose SCT early because the teratoma is of relatively small size. So routine antenatal ultrasonography examination is recommended for early detection and monitoring of complications like hydrops fetalis, tumor rupture, high output cardiac failure. Large tumors increase the risk of fetal demise due to cardiac failure resulting form arteriovenous shunting of blood.^4^ Majority of sacrococcygeal tumors in newborn are benign they are of concern for few reasons, one being the risk of congestive cardiac failure and fetal hydrops as tumor size increases, other for high risk of perinatal mortality.^5^ The Altman classification system has divided SCT into four types, based on anatomical distribution of tumor.^1^ Type I tumor has significant external component, type II has almost equal extra and intrapelvic component. Similarly Type III tumors have significant intrapelvic parts while type IV tumors are exclusively intrapelvic in location. Treatment of SCT depends on the nature of the tumor and time of diagnosis. Complete surgical excision of tumor is sufficient for benign tumor while complex modalities are employed for malignant tumors including surgery and chemotherapy.^6^ However whether tumor is benign or malignant, surgery is the initial choice of treatment supplemented by regular follow up examination.^2^ Alpha Feto protein is to be monitored until 3 years of age and per rectal examination is done until 9 months of age.^7^, ^8^ Fetoscopic surgery for SCT includes minimally invasive procedures such as radiofrequency ablation (RFA) and laser ablation. These techniques involve the use of specialized instruments and are guided by fetoscopy.^9^ In contrast, open surgical resection is generally reserved for postnatal management rather than in utero treatment.

This case is noteworthy due to several aspects that contribute to its rarity and complexity. SCTs are the most common congenital tumors in neonates, but their occurrence in older pregnancies, as seen here, is unusual.^10^ Furthermore, the benign course of the pregnancy until the discovery of the fetal mass at 31 weeks' gestation contrasts with the potential complications that can arise from such tumors, such as polyhydramnios or fetal hydrops.^11^ The diagnostic journey and management of SCT also distinguish this case. Regular antenatal follow‐up and detailed ultrasound monitoring allowed for early detection and characterization of the tumor, essential for planning appropriate management.^12^ The decision for a cesarean section at 37 weeks was likely influenced by the size and location of the tumor, aiming to minimize risks during delivery. The immediate postnatal period involved thorough clinical evaluation and diagnostic imaging, including CT scan and alpha fetoprotein testing, which guided further management.^13^

Surgical resection in the neonatal period, as performed in this case, is standard practice due to the potential for rapid growth and complications associated with SCTs.^14^ The procedure described—A complete excision with preservation of adjacent structures—highlights the meticulous surgical approach necessary to achieve favorable outcomes, including minimal blood loss and preservation of function.^15^

Histopathological examination revealed an immature teratoma, a finding that adds complexity given its potential for malignancy, necessitating long‐term follow‐up.^16^ The gradual normalization of alpha fetoprotein levels postoperatively is reassuring and consistent with expected outcomes following complete resection.^17^ Comparatively, while SCTs are relatively common in neonates, their occurrence in pregnancies over the age of 25 years is infrequent. Cases involving older mothers often present with additional clinical considerations related to maternal age and prenatal care.^18^

Comparing the presented case of SCT with similar reported cases, several consistent themes and strategies emerge alongside some notable differences. Each case underscores the critical role of prenatal diagnosis and meticulous monitoring in guiding management decisions. For instance, Khalek et al. emphasize the importance of early detection through prenatal ultrasound, facilitating multidisciplinary planning and intervention.^12^ This parallels the approach taken in our case, where regular antenatal follow‐up and detailed ultrasound assessments were pivotal in tracking tumor growth and preparing for delivery. Surgical intervention remains the mainstay of treatment across all cases, aiming for complete excision while preserving surrounding structures to minimize morbidity. Studies by Dunn and Laberge highlight the necessity of expertise in achieving successful surgical outcomes and reducing postoperative complications.^14^ The described surgical procedure in our case, involving a complete resection and coccygectomy, reflects these principles, ensuring adequate tumor removal while maintaining functional integrity.

Histopathological evaluation plays a crucial role in determining the nature of the tumor and guiding postoperative care. Similar to other cases, our patient's histopathological findings of an immature teratoma underscore the need for long‐term surveillance due to its potential for malignancy.^17^ Monitoring of alpha fetoprotein levels postoperatively, as described in our case, aligns with protocols aimed at detecting recurrence or residual disease early. While there are consistent strategies across cases, differences may arise in specific management decisions such as the timing of delivery. For instance, variations in gestational age at delivery in response to tumor characteristics and maternal‐fetal considerations illustrate the individualized approach required in SCT management.^13^

## 6 CONCLUSION

The management of SCT presents unique challenges necessitating a multidisciplinary approach. Early surgical intervention, guided by precise clinical insights, is crucial for achieving optimal outcomes. Beyond surgical expertise, ongoing vigilance and comprehensive follow‐up are essential to monitor for recurrence and ensure the holistic well‐being of the patient. This case underscores the importance of integrating clinical knowledge with compassionate care, emphasizing the continuous evolution of therapeutic strategies in the management of this complex condition.

## AUTHOR CONTRIBUTIONS

**Anish Luitel:** Conceptualization; writing – original draft; writing – review and editing. **Rasmita Poudel:** Writing – original draft. **Sajjad Ahmed Khan:** Writing – original draft. **Hiramani Pathak:** Supervision; writing – review and editing. **Surya Bahadur Parajuli:** Supervision; writing – review and editing. **Soorya Bhattarai:** Supervision; writing – review and editing. **Nitesh Yadav:** Writing – original draft.

## 7 FUNDING INFORMATION

The authors did not receive financial support from any organization for the submitted work.

## CONFLICT OF INTEREST STATEMENT

The authors declare that they have no competing interest.

## ETHICS STATEMENT

Not required for case reports.

## CONSENT

Informed, well written consent was obtained from patient parents for published author.

## Data Availability

Data generated/analyzed during this study is available from the corresponding author on reasonable request.
